# Bronchopulmonary Sequestration with Morbid Neonatal Pleural Effusion despite Successful Antenatal Treatment

**DOI:** 10.3389/fped.2017.00259

**Published:** 2017-12-04

**Authors:** Natalie Divjak, Sabine Vasseur Maurer, Eric Giannoni, Yvan Vial, Anthony de Buys Roessingh, Barbara E. Wildhaber

**Affiliations:** ^1^Department Woman-Mother-Child, University Center of Pediatric Surgery of Western Switzerland, Lausanne University Hospital, Lausanne, Switzerland; ^2^Department Woman-Mother-Child, Clinic of Neonatology, Lausanne University Hospital, Lausanne, Switzerland; ^3^Department Woman-Mother-Child, Clinic of Obstetrics, Lausanne University Hospital, Lausanne, Switzerland

**Keywords:** bronchopulmonary sequestration, pleural effusion, antenatal treatment, neonatology, pediatric surgery

## Abstract

**Introduction:**

Bronchopulmonary sequestration (BPS) may cause prenatal pleural effusion (PE) or even hydrops. This case describes a fetus presenting with severe PE, which prenatally waned completely under steroid treatment, yet surprisingly reappeared rapidly after birth, requiring early surgical intervention.

**Case description:**

A male fetus was diagnosed with left BPS and severe PE. After three courses of prenatal steroid therapy for each recurrence of PE from 27 weeks of gestation, we observed a complete regression of PE prenatally. Yet, PE recurred 18 h after birth and persisted after repeated drainages and steroid therapy. Early total resection of the extralobar BPS was performed and led to complete recovery without recurrence of PE.

**Conclusion:**

This report underlines that in cases of BPS presenting with prenatal PE needing fetal intervention, even if full regression of PE is observed before birth, there might be a need for surgical excision during the neonatal period.

## Introduction

Routine ultrasound screening during pregnancy usually may allow for the early detection of bronchopulmonary malformations. Various congenital lung lesions may present with associated complications, such as pleural effusion (PE), mediastinal shift, hydrops, and polyhydramnios. In the case of bronchopulmonary sequestration (BPS), up to one-third of fetuses present with severe PE or hydrops and require prenatal intervention (1, 2). Among these complications, hydrops has been shown to be the single prenatal outcome predictor for mortality (2, 3). Perinatal mortality concerns about 10% of babies presenting with BPS (2). Of note, 17% of infants born with BPS will present symptoms requiring emergency surgery during the first month of life (4). However, most of the infants will develop symptoms only later on, with an average time of onset of symptoms in prenatally detected lung lesions of about 7 months, thus only needing elective surgery during early infancy (4).

## Case Report

**Figure 1:**
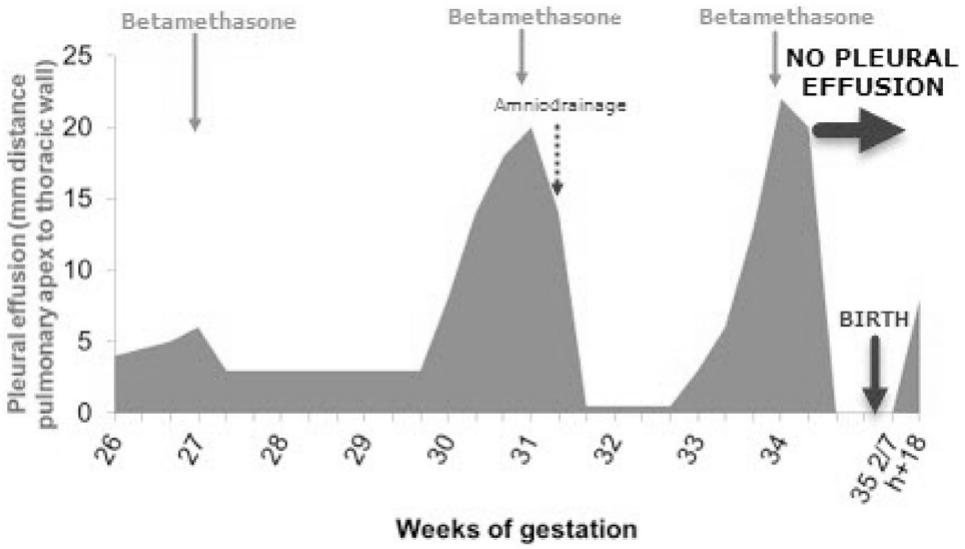
Prenatal course of pleural effusion (PE) and its treatment. In total, three courses of betamethasone were administrated, allowing for complete prenatal regression of the PE.

A 26-week-old male fetus presented with a pulmonary mass of 19 cm^3^, seen at standard ultrasound screening in our tertiary university hospital. A feeding vessel originating from the aorta was visualized and suggested the diagnosis of BPS. Weekly ultrasound monitoring was performed thereafter. At 27 weeks of gestation, moderate PE appeared, for which intramuscular betamethasone was administered to the mother. Ultrasound follow-up showed fluctuation of PE with good response to steroids at each recurrence. Three courses of betamethasone 2 mg/day for 2 days were administrated in total at 27 5/7, 31 2/7, and 34 1/7 weeks of gestation, with PE regression after each course. Amniodrainage was performed at 32 weeks for polyhydramnios (amniotic fluid index 44), because of maternal discomfort and risk of premature rupture of membranes, which yet recurred thereafter. At 34 weeks, when the thoracic lesion measured 23 cm^3^, scalp edema was observed concomitant with PE, however, followed by its complete regression after the third administration of betamethasone. Prenatal course of PE is summarized in Figure 1.

**Figure 2:**
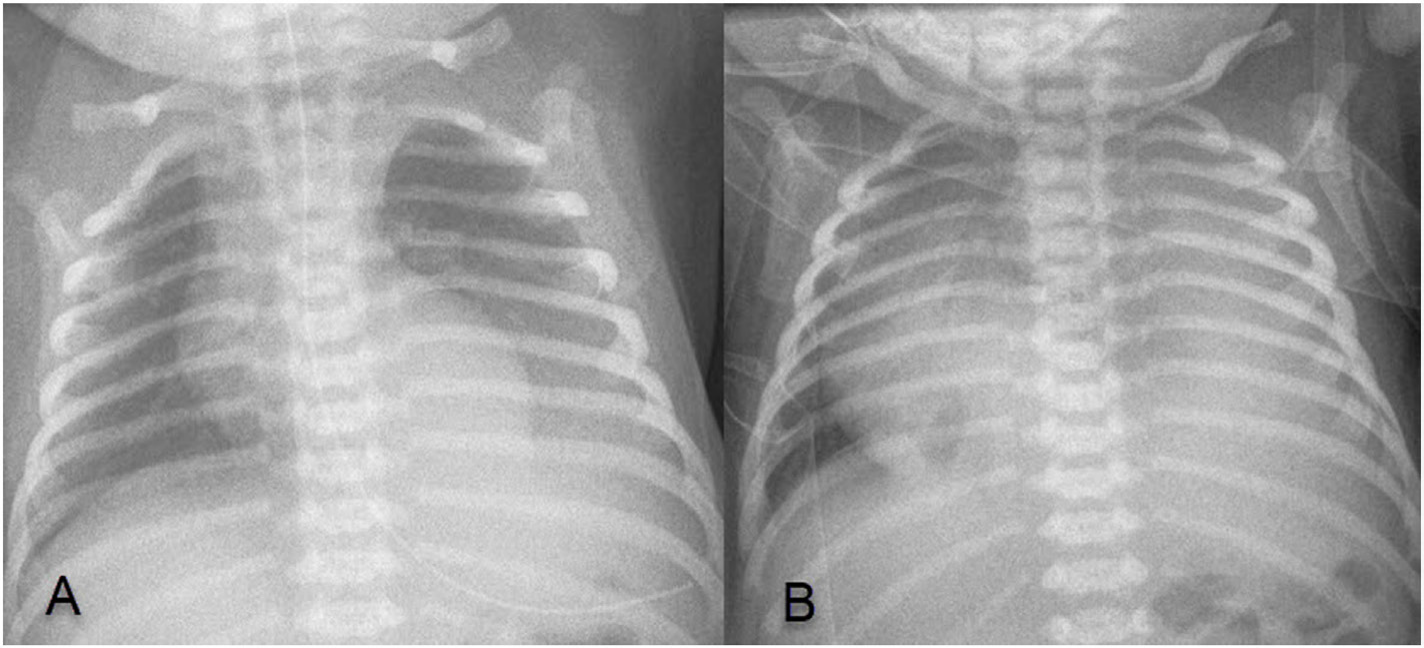
**(A)** X-ray 4 h after birth showing left basal thoracic mass, without significant pleural effusion (PE); **(B)** X-ray at 18 h of age showing a massive left sided PE causing mediastinal shift.

At 35 5/7 weeks, premature rupture of membranes occurred, probably due to recurrence of polyhydramnios and previous drainage. Cardiotocogram during labor was without pathological findings and vaginal delivery was uncomplicated. The newborn adapted well with Apgar score of 8/10/10. Birth weight was 2,570 g (P10–P50). There was no sign of respiratory distress or cardiovascular compromise. A first X-ray performed 4 h after birth showed a left basal thoracic mass without significant PE (Figure 2A). Yet, the newborn developed respiratory distress at the age of 18 h requiring treatment with high flow nasal cannula. X-ray showed massive left sided PE causing mediastinal shift (Figure 2B). Immediate pleural drainage allowed for clinical improvement. The analysis of the pleural fluid showed low protein and low leukocyte content compatible with transudate.

Persistent active pleural drainage during the following days lead to radiological work-up by ultrasound and CT-scan confirming the diagnosis of extralobar BPS, measuring 3.9 cm × 4.8 cm × 3.9 cm, with a feeding vessel originating from the aorta at the level of the diaphragm.

At 1 week of age, pleural drainage increased up to 80 ml/kg/day. Dexamethasone was administered for 1 week, at a decreasing dosage of 0.15 mg/kg/day for 3 days to 0.1 mg/kg/day, followed by complete resolution of PE, and thus removal of the chest tube. One day after discontinuation of the steroid treatment, PE recurred needing a new chest tube insertion. Early excisional surgery was then considered as inevitable, and at day 27 the extralobar BPS was removed by thoracotomy. Histological analysis confirmed diagnosis of BPS and extensive pleuropulmonary lymphatic dilatation. Post-operative course was uneventful with complete recovery within days.

## Discussion

The antenatal discovery of a cystic or solid lung malformation opens a range of diagnosis, with the most frequent being congenital pulmonary airway malformation and BPS. Recent studies show that even the distinction between these two entities is not so clear with the discovery of an increasing number of “hybrid” lesions (3). Natural fetal evolution of these lesions may either tend toward enlargement, or to steady state, or even to partial/complete regression (3). When presenting antenatal complications, such as massive PE, with or without mediastinal shift, or even hydrops, fetal intervention may be required (5). Pleural–amniotic shunting is an option in these cases. BPS offers more specific therapeutic options by acting on the feeding vessel by laser, radiofrequency, or embolization. A recent study comparing pleuroamniotic drainage to laser ablation of the feeding vessel showed a tendency toward an advantage for ablation of the feeding vessel, even though numbers are too small to demonstrate a definitive advantage (1). In the case of microcystic lesions, maternal steroid treatment with betamethasone has shown to be a safe and efficient by reducing hydrops and also the size of the lesions. Of note, no adverse maternal or fetal events resulting from betamethasone administration have been reported (6). It is believed that the administration of steroids allows for lung maturation in these bronchopulmonary malformations, which are considered as an arrested state of development. Yet, the role of maternal betamethasone treatment in BPS has not been clearly established due to their relative lower incidence (7). In our case, where steroid administration was chosen, the fetus responded to repeated courses of betamethasone with complete regression of PE and decrease in size of the BPS.

Because there is a twofold increase in risk of operative complications in symptomatic patients, elective surgery is aimed to be performed before appearance of symptoms, such as respiratory distress or infection (4). Average time of onset of symptoms is reported to be at the age of about 10 months, taking into consideration both infants diagnosed prenatally and those who were not (4). Thus, most pediatric surgeons generally plan elective surgery in infants presenting with congenital lung lesions at the age of 6–12 months (8). The reported neonate was completely asymptomatic at birth, and also once the postnatally recurrent PE was drained, allowing time for observation of natural evolution of the PE. In addition, we attempted a conservative treatment with corticosteroids, since this was effective during pregnancy. Response was favorable, but did not avoid recurrence of PE. We then chose to perform surgery to avoid prolonged pleural drainage, with the risk of infection, as well as long-term steroid treatment, associated with its well-known side effects.

To the best of our knowledge, the postnatal recurrence of PE after complete antenatal regression has not been described in the literature. The reason for postnatal recurrence of PE remains unclear. Reports suggest that severe PE might be due to obstruction of venous return due to compression by the BPS, cardiac failure due to shunting through the lesion or obstruction of lymphatic return. The chemical and cytological analysis of the pleural liquid, compatible with transudate, is in line with this hypothesis. Histological examination in our case showed marked lymphatic dilatation, a potential explanation for recurrent PE in our case: steroids may have influenced the local lymphatic tissue, yet without persistent effect.

## Concluding Remarks

Antenatal ultrasound scan is a sensitive tool, which offers early detection of BPS and its antenatal complications, and thus guides intervention. These measures may lead to complete antenatal resolution of PE. Nevertheless, this case illustrates that, despite a favorable antenatal course, there is a probability that surgical intervention during the neonatal period still might be required due to recurrence of PE after birth.

## Ethics Statement

Parental written consent was obtained.

## Author Contributions

ND and BW were responsible for the initial written content as well as editing the manuscript and creating the image. All other authors were responsible for reviewing and editing the manuscript in its intellectual content as well as its form; gave their final approval for the version to be published.

## Conflict of Interest Statement

The authors declare that the research was conducted in the absence of any commercial or financial relationships that could be construed as a potential conflict of interest.

## Abbreviations

BPS, bronchopulmonary sequestration; PE, pleural effusion.
